# Analysis of the melon (*Cucumis melo*) small RNAome by high-throughput pyrosequencing

**DOI:** 10.1186/1471-2164-12-393

**Published:** 2011-08-03

**Authors:** Daniel Gonzalez-Ibeas, José Blanca, Livia Donaire, Montserrat Saladié, Albert Mascarell-Creus, Ana Cano-Delgado, Jordi Garcia-Mas, Cesar Llave, Miguel A Aranda

**Affiliations:** 1 Departamento de Biología del Estrés y Patología Vegetal, Centro de Edafología y Biología Aplicada del Segura (CEBAS) - CSIC, Apdo. correos 164, 30100 Espinardo (Murcia), Spain; 2 Departamento de Biotecnología, Instituto de Conservación y Mejora de la Agrodiversidad Valenciana (COMAV) - UPV, Camino de Vera s/n, 46022 Valencia, Spain; 3 Departamento de Biología Medioambiental, Centro de Investigaciones Biológicas (CIB) - CSIC, Ramiro de Maeztu 9, 28040 Madrid, Spain; 4 IRTA, Center for Research in Agricultural Genomics CSIC-IRTA-UAB, Campus UAB, Edifici CRAG, Bellaterra (Cerdanyola del Vallès), 08193 (Barcelona), Spain; 5 Molecular Genetics Department, Center for Research in Agricultural Genomics (CRAG) CSIC-IRTA-UAB, Campus UAB, Edifici CRAG, Bellaterra (Cerdanyola del Vallès), 08193 (Barcelona), Spain

## Abstract

**Background:**

Melon (*Cucumis melo *L.) is a commercially important fruit crop that is cultivated worldwide. The melon research community has recently benefited from the determination of a complete draft genome sequence and the development of associated genomic tools, which have allowed us to focus on small RNAs (sRNAs). These are short, non-coding RNAs 21-24 nucleotides in length with diverse physiological roles. In plants, they regulate gene expression and heterochromatin assembly, and control protection against virus infection. Much remains to be learned about the role of sRNAs in melon.

**Results:**

We constructed 10 sRNA libraries from two stages of developing ovaries, fruits and photosynthetic cotyledons infected with viruses, and carried out high-throughput pyrosequencing. We catalogued and analysed the melon sRNAs, resulting in the identification of 26 known miRNA families (many conserved with other species), the prediction of 84 melon-specific miRNA candidates, the identification of *trans-*acting siRNAs, and the identification of chloroplast, mitochondrion and transposon-derived sRNAs. *In silico *analysis revealed more than 400 potential targets for the conserved and novel miRNAs.

**Conclusion:**

We have discovered and analysed a large number of conserved and melon-specific sRNAs, including miRNAs and their potential target genes. This provides insight into the composition and function of the melon small RNAome, and paves the way towards an understanding of sRNA-mediated processes that regulate melon fruit development and melon-virus interactions.

## Background

Melon (*Cucumis melo *L., family *Cucurbitaceae*) is an important horticultural species cultivated in temperate, subtropical and tropical regions worldwide, with Spain being the largest producer in Europe and fifth in the world [1]. The melon genome has 12 chromosomes and is thought to contain 450-500 Mb of DNA, which is 3-4 times more than Arabidopsis [2]. Melon is a useful model for the analysis of fruit traits because of the vast morphological, physiological and biochemical diversity within the species, which can be exploited to dissect the biological processes controlling color, flavor and texture and how these properties arise during fruit development [3,4].

Despite the importance of melon, not much was available in the way of genomic sequence information prior to the establishment of a functional genomics consortium in 2004, which developed a range of tools and accumulated more than 33,000 expressed sequence tags (ESTs) and ~17,000 tentative consensus sequences (unigenes) [5]. This EST collection has been expanded recently with the addition of 94,000 new ESTs from full-length enriched cDNA and standard cDNA libraries from various melon tissues and cultivars in the framework of the International Cucurbit Genome Initiative [6]. These ESTs as well as other resources are now accessible in a public database [7]. The unigene sequences have also been used to construct an oligonucleotide microarray, which has been applied in the analysis of fruit quality traits, ovary development and pathogen resistance [8]. In addition, a melon sequencing consortium has recently produced a high-quality draft of the melon genome (unpublished data). Although these resources provided significant advances in the analysis of melon gene expression, the small RNA (sRNAs) component of the melon transcriptome has not been studied in detail. These important molecules have been studied in other crop species and have been shown to fulfill a number of critical regulatory roles [9-12].

sRNAs are short, non-coding RNAs 21-24 nucleotides (nt) in length which are found in protists, fungi, plants and animals [13]. In plants, their roles include maintenance of genome stability, initiation of heterochromatin assembly, post-transcriptional regulation of gene expression and protection against viruses using an RNA-based immune system. The most abundant and best-characterised sRNAs include microRNAs (miRNAs) and small interfering RNAs (siRNAs). miRNAs are widely studied because of their regulatory activity, particularly in development, pathogen resistance and stress responses [13]. miRNAs are cleaved from stem-loop precursor molecules that derive from single stranded non-coding transcripts. miRNAs regulate protein-coding genes post-transcriptionally by mediating RNA cleavage or translational repression. Unlike miRNAs, siRNAs are generated from double-stranded RNA precursors and function on cognate RNA or DNA molecules by instigating degradation or promoting RNA-directed DNA methylation, respectively. cis-acting siRNAs (ca-siRNAs) arise from and target endogenous loci such as transposons and DNA repeats to direct cytosine methylation and chromatin modifications [14]. Natural antisense-transcript siRNAs (nat-siRNAs), which derive from pairs of natural-antisense transcripts, guide the cleavage of one of the two parent transcripts, leading to the production of a series of secondary 21-nt siRNAs of unclear function [15,16]. Finally, trans-acting siRNAs (ta-siRNAs) derived from *TAS *genes, which transcribe long primary non-coding RNAs as precursors for ta-siRNA biogenesis. TAS primary RNAs are cleaved by specific miRNAs and are sequentially processed into 21-nt ta-siRNAs starting from the miRNA-cleaved end, to generate clusters of phased siRNAs [17,18]. In addition to endogenous sRNAs, exogenous siRNAs from virus genomes can be detected in virus-infected plants as a part of the RNA-based immune system [19].

RNA viruses that infect melon are responsible for significant yield losses as well as poor fruit quality [20,21], particularly the widespread *Watermelon mosaic virus *(WMV, genus *Potyvirus*, family *Potyviridae*) [22,23]. Recently, a collection of accessions representing cultivated melon and its wild relatives was screened to identify sources of resistance to mosaic-inducing viruses [24]. TGR-1551 was identified as a resistant accession based on the lower WMV titer compared to susceptible genotypes (e.g. melon cv. Tendral) and the absence or mildness of the mosaic symptoms normally observed in systemically infected leaves [25]. *Melon necrotic spot virus *(MNSV, genus *Carmovirus*, family *Tombusviridae*), although less economically important, may also cause yield losses, and epidemic outbreaks have been reported worldwide [26,23]. In melon, resistance to MNSV is controlled by the single recessive gene *nsv*, which encodes eukaryotic translation initiation factor 4E (Cm-eIF4E) [27]. This resistance is effective against all MNSV strains (e.g. MNSV-Malfa5) except MNSV-264 [28]. Studies of chimeric viruses have shown that the MNSV 3' untranslated region (3'-UTR) contains the resistance-breaking determinant of MNSV-264, and that it functions as a cap-independent translational enhancer [29,30].

We constructed 10 sRNA libraries from a range of healthy and virus-infected melon tissues, and we sequenced a set of endogenous and exogenous sRNAs using the pyrosequencing-based 454 technology from Roche [31]. To gain insights into the role of sRNAs on key aspects of fruit development, maturation and pathogen defense, samples from two stages of the developing ovary, fruits 15 and 45 days after pollination, and photosynthetic cotyledons from resistant and susceptible melon accessions infected with WMV and MNSV were analysed. In a previous study, we reported the profile of virus-derived sRNAs (viRNAs) from cotyledon samples [32]. Here we report a catalog of endogenous melon sRNAs, including miRNAs from known families and new candidate miRNAs potentially unique to melon, focusing on the number of sequence reads as a reflection of their expression profiles. Potential targets for these miRNAs in the melon transcriptome were identified.

## Results

### cDNA libraries and sequencing of small RNAs

**Figure 1:**
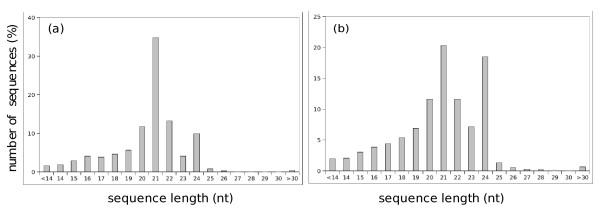
**Representation of sequences with different lengths in the melon sRNA data set**. *(a) *All sequences. *(b) *Non-redundant sequences. The number of sequences is expressed as a percentage of the total number of sequences.

We used high throughput sequencing data to analyze the composition of the small RNA transcriptome (sRNAome) of melon and compare the results to data in publicly-available RNA and genomic databases. Ten sRNA libraries were constructed from total RNA extracted from fruits, ovaries and healthy and virus-infected melon cotyledons (Table 1). PCR amplification products corresponding to each library were pooled in equal amounts and sequences were obtained by multiplexed high-throughput pyrosequencing (Roche 454). This produced 447,180 raw sequences, each ~100 bases in length, 432,743 of which had a complete 3' adaptor in the correct position. Based on these data, we estimated a sequencing error rate of 3.7%. After removing reads where one or the two adaptors could not be identified, 398,450 useful sequences with 3' and 5' adaptors were selected. Only 44 sequences comprising ligated adaptors without an insert were identified. Although we pooled similar amounts of PCR products from each library, different numbers of sequences were obtained according to the 5' adaptor sequence barcode (Table 1). For instance, the fruit and ovary libraries (15d, 45d, c1 and c5) were poorly represented providing a collection of fewer than one third of the number of sequences from the other six libraries. A set of 186,698 non-redundant sRNA sequences was generated for downstream analysis. The representation of sequences with different lengths in the redundant and non-redundant sRNAs datasets is shown in Figure 1. The most abundant sequences were 21, 24, 20 and 22 nts. A few sequences shorter than 20 nt were also retrieved, and these probably represent cloning artifacts and/or degradation products. Sequences > 30 nt in length in our dataset predominantly represented combinations of other melon sRNAs identified in our work. Detailed data are provided in Additional file 1.

**Table 1:** Description of small RNA libraries from different melon tissues

Library	Cultivar/accession	Tissue	Physiological condition	**Virus**^**a**^	Reads	Unique sequences
Wtm	cv. Tendral	Cotyledon	Mock-inoculated	--	33123	15624
Wt	cv. Tendral	Cotyledon	Virus-infected	WMV-M116	35860	12840
Cwm	accession TGR-1551	Cotyledon	Mock-inoculated	--	41039	21122
Cw	accession TGR-1551	Cotyledon	Virus-infected	WMV-M116	36330	24100
15d	cv. Piel de Sapo	Fruit	Healthy, 15 days after pollination	--	21662	14620
45d	cv. Piel de Sapo	Fruit	Healthy, 45 days after pollination	--	9942	8167
c1	cv. Piel de Sapo	Ovary	Healthy	--	18764	15269
c5	cv. Piel de Sapo	Ovary	Healthy	--	14529	12608
Ta5	cv. Tendral	Cotyledon	Virus-infected	MNSV-alfa5	43170	22869
3'T	cv. Tendral	Cotyledon	Virus-infected	MNSV (chimeric)	56425	56425

^a^WMV = *Watermelon mosaic virus*; MNSV (alfa5) = *Melon necrotic spot virus*, alfa5 isolate; MNSV (chimeric) = *Melon necrotic spot virus*, alfa5 isolate with 3' UTR from 264 isolate

### Identification of known miRNAs

In order to identify known miRNAs, the melon sRNA data set was used as a BLAST query against the Arabidopsis small RNA database (ASRP) [33] and the microRNA database (miRbase) [34]. We identified 46 melon unique sequences corresponding to 26 miRNA families. Thirty nine sequences were identical to known miRNAs from other plant species, while 7 additional species were sequence variants highly conserved (up to two mismatches allowed). In order to clearly identify each melon sequence, melon miRNAs were named according to the homologous reference miRNA from each database (Table 2). For each reference miRNA, we found that ~3% of the corresponding melon sequences differed at one or two sites with mismatches distributed randomly along the sequence, so these were considered sequencing errors. Only specific sequence variants that represented more than 3% of the total population for each reference miRNA were considered biologically relevant. We identified only two non-conserved miRNAs, corresponding to ath-miR2111a from Arabidopsis and peu-miR2910 from *Populus euphratica*, respectively (Table 2). The largest diversity of miRNA species was found in ovary samples and the lowest in fruit samples.

**Table 2:** Known plant miRNAs identified in melon

Annotation	Melon sRNA sequence (5'-3')	Similarity	Number of miRNA sequences	miRNA* sequences	**Hit in melon genome**^**a**^
miR156|a, b, c, d, e, f	UGACAGAAGAGAGUGAGCAC	100%	469	60	YES
miR157|a, b, c	UUGACAGAAGAUAGAGAGCAC	100%	269	0	YES
miR157|d	UGACAGAAGAUAGAGAGCAC	100%	19	21	YES
miR158|a	UCCCAAAUGUAGACAAAGCA	100%	1	0	--
miR159|a	UUUGGAUUGAAGGGAGCUCUA	100%	14651	0	YES
miR159|b	UUUGGAUUGAAGGGAGCUCUU	100%	18	0	--
miR159|c	UUUGGAUUGAAGGGAGCUCCU	100%	1	0	--
miR160|a, b, c	UGCCUGGCUCCCUGUAUGCCA	100%	537	0	YES
miR161|a.1	UUGAAAGUGACUACAUCGGGG	100%	6	0	--
miR161|a.2	UCAAUGCAUUGAAAGUGACUA	100%	1	0	--
miR162|a, b	UCGAUAAACCUCUGCAUCCAG	100%	825	0	YES
miR164|a, b	UGGAGAAGCAGGGCACGUGCA	100%	172	1	YES
miR165|a, b	UCGGACCAGGCUUCAUCCCCC	100%	4	0	--
miR166|a, b, c, d, e, f, g	UCGGACCAGGCUUCAUUCCCC	100%	65	27	YES
miR167|a, b	UGAAGCUGCCAGCAUGAUCUA	100%	136	0	YES
miR167|d	UGAAGCUGCCAGCAUGAUCUGG	100%	16	1	--
miR168|a, b	UCGCUUGGUGCAGGUCGGGAA	100%	967	0	YES
miR169|a	CAGCCAAGGAUGACUUGCCGA	100%	3	1	--
miR169|b, c	CAGCCAAGGAUGACUUGCCGG	100%	76	1	YES
miR169|h, i, j, k, l, m, n	UAGCCAAGGAUGACUUGCCUG	100%	83	1	YES
miR170|a	UGAUUGAGCCGUGUCAAUAUC	100%	3	0	--
miR171|a	UGAUUGAGCCGCGCCAAUAUC	100%	85	5	YES
miR171|b, c	UUGAGCCGUGCCAAUAUCACG	100%	64	0	YES
miR172|a	AGAAUCUUGAUGAUGCUGCAU	100%	85	58	YES
miR172|c, d	AGAAUCUUGAUGAUGCUGCAG	100%	4	0	YES
miR172|e	GGAAUCUUGAUGAUGCUGCAU	100%	3	0	YES
miR319|a, b	UUGGACUGAAGGGAGCUCCC	100%	2	3	YES
miR390|a, b	AAGCUCAGGAGGGAUAGCGCC	100%	32	6	YES
miR391|a	UUCGCAGGAGAGAUAGCGCCA	100%	1	0	--
miR393|a, b	UCCAAAGGGAUCGCAUUGAUC	100%	18	0	YES
miR394|a, b	UUGGCAUUCUGUCCACCUCC	100%	4	0	YES
miR396|a	UUCCACAGCUUUCUUGAACUG	100%	134	84	YES
miR396|b	UUCCACAGCUUUCUUGAACUU	100%	82	16	YES
miR397|a	UCAUUGAGUGCAGCGUUGAUG	100%	26	0	YES
miR408|a	AUGCACUGCCUCUUCCCUGGC	100%	14	1	YES
ath-miR2111a	UAAUCUGCAUCCUGAGGUUUA	100%	1	0	YES
peu-miR2910	UAGUUGGUGGAGCGAUUUGUC	100%	8	0	YES
osa-miR167d	UGAAGCUGCCAGCAUGAUCUG	100%	3401	1	YES
tae-miR395b	UGAAGUGUUUGGGGGAACUC	100%	1	0	YES
bna-miR397a	CAUUGAGUGCAGCGUUGAUGU	95%	77	0	YES
miR156|h	UUGACAGAAGAGAGUGAGCAC	95%	91	0	YES
miR156|g	ACAGAAGAGAGUGAGCACA	90%	5	0	YES
miR169|d, e, f, g	UGAGCCAAGGAUGACUUGCCU	95%	130	0	YES
miR169|d, e, f, g	UGAGCCAAAGAUGACUUGCCU	90%	112	0	YES
miR399|a	UGCCAAAAGAGACUUGCCCUG	95%	3	0	YES
miR403|a	CUAGAUUCACGCACAAGCUCG	90%	1	0	--

^a ^Sequences with hit in melon genome = 'YES'; sequences with no hit = '--'

**Figure 2:**
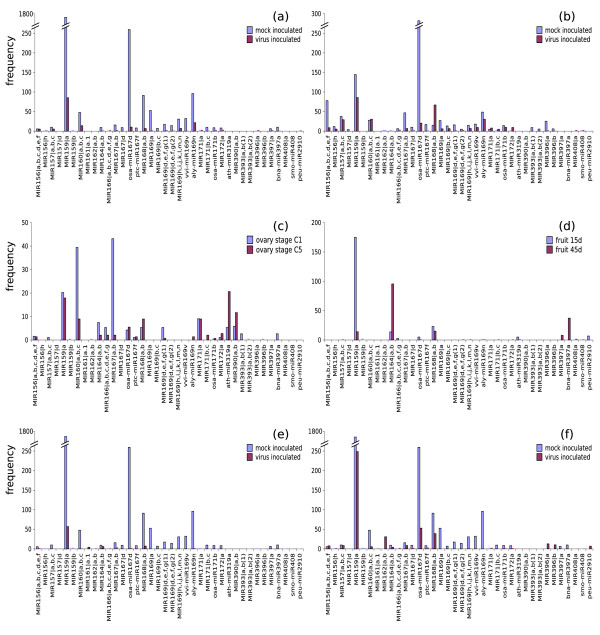
**Relative accumulation of conserved miRNAs in melon samples used for sRNA library construction**. Total reads for each miRNA in each library were normalised relative to the total number of reads from the library, and expressed per 10,000 reads. *(a) *Cotyledons from melon cv. Tendral inoculated with WMV-M116 compared to mock inoculated cotyledons of the same cultivar. *(b) *Cotyledons from the melon accession TGR-1551 inoculated with WMV-M116 compared to mock inoculated cotyledons of the same accession. *(c) *Stage C1 and C5 ovaries from melon cv. Piel de Sapo. *(d) *Fruit from melon cv. Piel de Sapo 15 days after pollination (15d) compared to fruit from the same cultivar 45 days after pollination (45d). *(e) *Cotyledons from melon cv. Tendral inoculated with MNSV-alfa5 compared to mock-inoculated cotyledons of the same cultivar. *(f) *Cotyledons from melon cv. Tendral inoculated with MNSV (chimeric virus) compared to mock inoculated cotyledons of the same cultivar.

The abundance distribution of different miRNAs in each library was estimated based on sequencing frequencies as shown in Figure 2. We used sequencing data for quantitative profiling of small RNAs, though estimation of abundance based on sequencing frequencies could be misleading due to limited sequencing depth. Many miRNAs differed in abundance according to the source library. Nevertheless, most of the redundancy reflected the accumulation of miR159a, which accounted for more than 14,000 sequences in total. Figure 2A, B compares the accumulation of miRNAs in healthy *versus *WMV-infected melon tissues from genotypes Tendral and TGR-1551. Melon miRNA species with similarity to Arabidopsis miR156abcdef, miR160abc and miR168ab, which target mRNAs encoding squamosa promoter binding proteins, auxin response factors (ARFs) and argonaute-like proteins (AGO), respectively, showed different trends in the genotypes tested. For example, miR168ab is more abundant in healthy Tendral tissues compared to infected tissues whereas it is more abundant in WMV-infected TGR-1551 tissues than in healthy tissues. Other known miRNAs in our sequenced set were generally more abundant in healthy tissues irrespective of the melon variety tested. For example, miRNAs with similarity to Arabidopsis miR159a and miR167d, which target MYB transcription factors and ARFs, respectively, followed this trend in both genotypes albeit with differences in magnitude. Comparison of the two libraries from ovary and fruit samples (Figure 2C, D) revealed that miRNAs were particularly abundant and diverse in ovaries compared to fruits. Several miRNAs appeared to be temporally regulated during ovary development (e.g. members of the miR160, miR164, miR167, miR169, miR319 and miR390 families) whereas others were equally abundant at both ovary stages (miR156, miR167 and miR171 families). Fruits contained far fewer miRNAs than ovaries, and only miRNAs similar in sequence to Arabidopsis miR159a, miR164ab and miR397a showed significant differences in accumulation (with trends opposite to those seen in ovaries). These findings indicated that miRNAs in melon were expressed in specific tissues and in response to particular physiological conditions. In Arabidopsis, most of these miRNAs target mRNAs encoding transcription factors with roles in development, such as hormone signal transduction and organ identity. Figure 2E, F compares the accumulation of miRNAs in healthy and MNSV-infected tissues. Similar accumulation profiles were observed in both samples for most of the miRNAs identified. Exceptionally, miRNAs similar to Arabidopsis miR396a, miR396b and miR162a, which regulate transcripts encoding GRF transcription factors and DCL proteins, respectively, showed opposite accumulation patterns.

### Identification of miRNA/miRNA* duplexes

**Figure 3:**
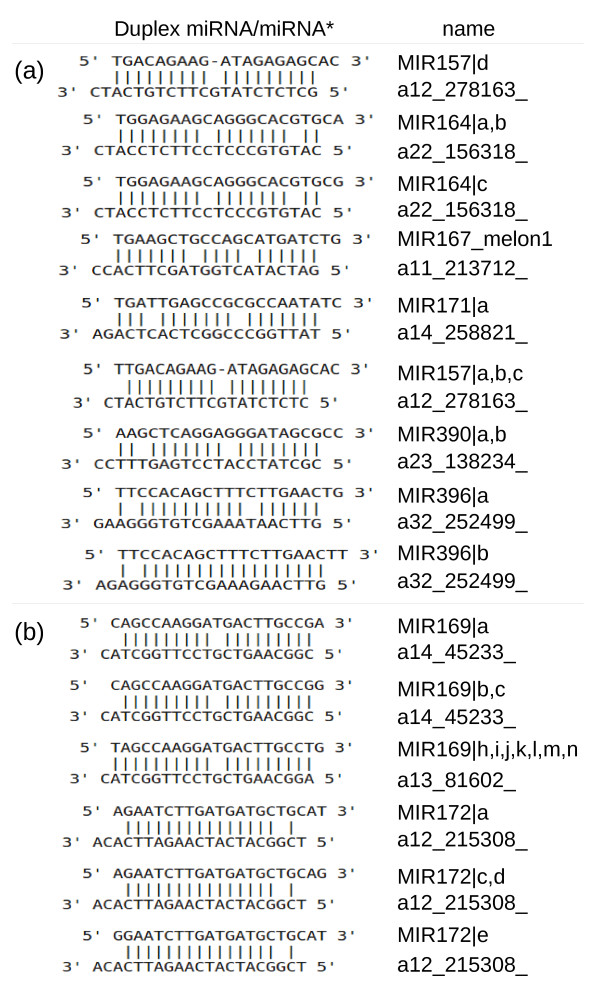
Duplexes of mature miRNA and passenger (miRNA*) sequences identified in the melon sRNA collections. *(a) *Typical duplex structure. *(b) *Non-typical duplex structure (number of protruding nucleotides ≠ 2).

DCL-mediated cleavage of miRNA precursors having the characteristic stem-loop structure gives rise to miRNA duplexes where one of the two strands is the guide miRNA (the functional molecule) while the near-perfect complement sequence is known as the passenger miRNA, or miRNA*. The miRNA* is rapidly degraded but transient species can be cloned and therefore sequenced. We identified 16 miRNA* sequences complementary to some of the 46 miRNAs in our dataset (Table 2), nine of which had the predicted sequence based on the fold-back structure of their presumptive precursors with internal mismatches and two additional terminal nucleotides forming a 3' tail (Figure 3A), whereas the other six had a different number of protruding nucleotides and were considered non-typical (Figure 3B).

The number of sequenced miRNA*s was generally much lower than the number of mature sequences but there were some remarkable exceptions. For example, for miR396a we counted 134 miRNA and 84 miRNA* sequences, as opposed to miR159a for which 14,651 miRNA sequences but no corresponding miRNA* sequences were retrieved in the sequenced collections (Table 2). The most extreme example was miR157d, for which we recovered the same numbers of miRNA and miRNA* sequences.

### Identification of putative melon-specific miRNAs

After identification of known miRNA sequences and other sRNA sequences (see below), 108,454 unique melon sRNAs remained unclassified, from which the most abundant (28.6%) were 24-nt species. Initial analysis confirmed that 36,783 (33.9%) of these sequences had a perfect match in the melon genome. The frequency distribution was highly skewed: 33,621 sequences had fewer than 25 hits (24,488 originated from a single locus), and only 659 sequences had more than 100 hits.

Sequences that were 21, 22 or 24 nt in length with a maximum of six hits in the genome were selected as potential novel miRNAs, and flanking genomic regions were analysed according to three consecutive criteria. First, we used miRanda software to detect sequences complementary to the potential miRNA inside the flanking regions. Second, potential miRNAs with precursors less than 70 nt in length were discarded. Finally, the MFEI index, which is used to distinguish miRNA precursors from other coding and non-coding RNAs and is based on free energy estimates and nucleotide composition [35], was calculated for each precursor and the results were sorted accordingly (the more negative the index, the better the precursor).

**Figure 4:**
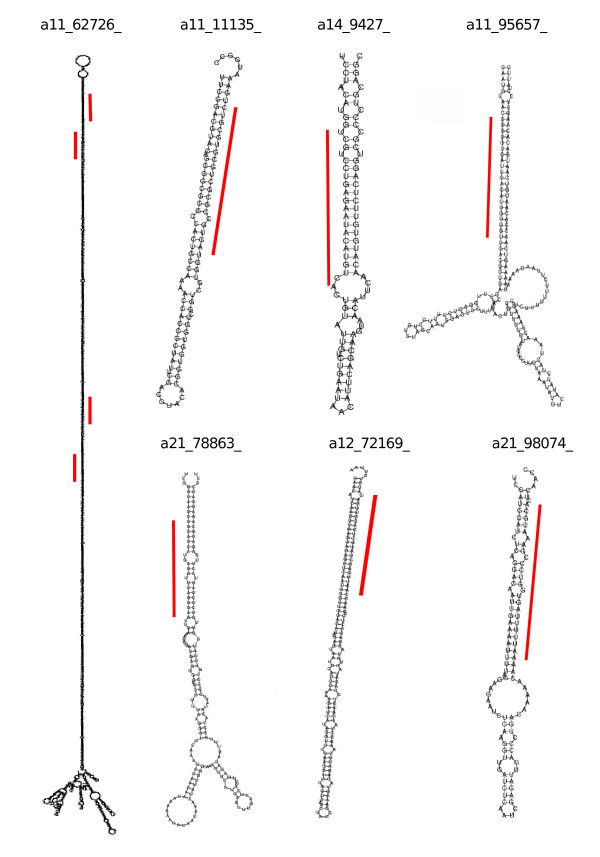
**Secondary structures of putative novel melon-specific miRNA precursors**. Some of the sRNAs identified as potentially novel and melon-specific miRNAs (listed in Table 3) were selected based on quality criteria and are shown as examples. Red lines represent regions where miRNA/miRNA* duplexes are located. Identifier a11_72726 represents an example of unsuitable miRNA candidate.

Predicted miRNA precursors and their genomic flanking regions that were found to be similar in sequence to previously described transposons were discarded. Other predicted miRNA precursors with intramolecular folding potential showed no similarity to known transposon sequences although their secondary structures were similar to those of known foldback transposons; these were characterised by strong negative MFEI indexes and high miRanda scores, both features consequence of high sequence complementarity in the pairing stem sequence. For some of these precursors, several uncharacterised melon sRNAs mapped on them in both the sense and antisense orientations (e.g. a11_62726 in Figure 4), up to 85 in some cases. Therefore, these were also considered unsuitable miRNA candidates. Three other potential miRNAs were shown to be the miRNA* sequences of known miRNAs that had not been picked up in our initial screen.

After manually inspecting the remaining secondary structures, 77 loci that fulfilled the structural criteria for annotation of plant miRNAs [36,37] were selected as plausible miRNA precursors; we also added to this list 7 other loci that had an asymmetric bulge involving 3 bases inside the putative miRNA duplex. From them, 43, 20 and 21 corresponded to sequenced sRNAs of 21, 22 and 24 nt in length, respectively (Table 3). Six selected sequences are shown as examples in Figure 4. By checking the pairing sequence on the stem of the predicted precursors, miRNA*s for seven candidate miRNAs were found in the sequenced set. Therefore these miRNAs were regarded as authentic miRNAs that conformed to the biogenesis and expression criteria for confident miRNA annotation [37]. The remaining sequences, not supported by the complementary passenger strands, were classified as candidate miRNAs. Most of the potential novel miRNA were represented by a small number of sequences, a single sequence in more than half of the cases, but six exceptional candidates were represented by more than 10 sequences (Table 3). As occurred for conserved miRNAs, sequence variants were identified for some novel miRNAs (Table 3) which mapped on the genomic melon sequence with slight variations in length and position relative to the most abundant sequence. In the absence of a reference sequence from any database, these variants were counted. Sequencing errors of differential cleavage of potential miRNA precursors possibly explain these length and positional polymorphisms.

**Table 3:** Potential novel melon specific miRNAs

**Melon sRNA name**^**a**^	nt	sRNA sequence	Number of sequences	Hits in genome	**miRanda score**^**b**^	Potential precursor lenght (nt)	**MFEI**^**c**^
a34_130677_	21	AUAGAUAUUGAUAUGCUUUUA	1	4	163	94	-1.0573
a24_2602_	21	UGCUACAUGGUUUAUCAGUGA	2	5	115	72	-1.2524
a24_177791_	21	UCGCAGAAGAGAUGGCGCCGA	7	1	143	91	-0.8587
a23_118111_	21	CAUUGAUAGACACUAAUAGAA	1	5	167	90	-1.475
a33_14294_	21	AUAGACUUCUAUUGGUGUCUA	1	1	154	74	-1.5105
a32_31625_	21	GUUCCCACGGUAAUGAUAAUA	2	1	127	72	-1.1045
a32_1324_	21	AGGUGUCAUCUUGCUGCGAUA	1	1	179	99	-1.2
a22_190223_	21	UGUUAUGCAUGGCGUCGGGAG	1	5	187	157	-1.2915
a14_98657_	21	AUAGCGAAGUAUAUCAGUGAU	1	1	154	127	-1.2423
a14_51701_	21	UGAGCCGUGCCAAUAUCGACG	1	1	159	97	-1.1
a14_180374_	21	UAAAUAUUUAGAAAGUCAAUC	1	4	159	79	-1.855
a21_80766_^f^	21	UAAUAUUCAUUUUCACUUCUU	1	1	116	232	-1.2494
**a21_78863_**	21	UGCAUCCUGAGGUUUAGGGAG	3	1	159	194	-0.9776
a21_244426_	21	AGUAACCACUAAGCUAAUGGC	1	1	187	597	-1.0096
a23_1441_^f^	21	UAUAGCAAAGUCUAUCGAUGG	5^d^	1	107	77	-1.1435
a13_84447_^e^	21	GGUCAUUCUAGCAGCUUCAAU	13^d^	1	139	201	-0.96
a23_370_^e^	21	UGGUGUGCAUGUGAUGGAAUA	13^d^	1	163	113	-0.9061
a21_134553_^f^	21	GUUAUACGAGUUGGGUUGGGU	1	1	155	114	-0.9571
**a11_95657_**^e^	21	UUGUGUCAUUGACAUUGUGGU	1	2	195	191	-1.1708
**a14_9427_**	21	UCGUCCUGAGAAUACAUGUCA	35^d^	1	159	97	-0.9409
a14_668_	21	UGAGUUAUCGGUGAAUUCAAG	5^d^	3	159	516	-0.9675
a13_252112_	21	ACUGCUGCUUGUACUAUUGAA	1	1	191	255	-1.9670
a11_33177_	21	UUUAGUUUAGCCUAUUGCUUU	1	3	187	139	-1.1035
a11_191362_^e^	21	UUCUAUUGUCUUCAUUUGUGA	1	1	191	119	-1.2718
a34_224062_	21	UGAAAUGACUUGUCAAGUGCU	1	1	151	97	-0.975
a13_228150_	21	CUUGUACUUGAUUUUGUUGCC	1	1	191	115	-1.4469
a12_32299_^e^	21	AAUUUGUUGGUCAAAUGAUUG	2	1	195	107	-1.7552
a12_272161_	21	UUGUAUGGUGGAAAGAUGGAA	1	1	162	96	-1.4167
a11_33986_	21	GCUGACUUGCUGAUUGAGUUA	2	3	179	189	-1.4852
a13_33760_	21	UGAAUUAUCUGCUUAAGUUUU	1	1	187	95	-1.2889
a11_389198_	21	ACACGCAGAAGAGACGAUUGA	1	1	191	120	-1.5575
a13_357842_	21	UGGAGCAAUAUUGAUGCAUAU	1	1	195	220	-0.9548
a11_364692_	21	UUGGGUCUAUUUAAUGGGAGC	1	1	155	107	-1.2308
a13_281334_	21	ACUUUCUGUCAAUAUAAUCAG	1	1	175	115	-1.3943
a12_71107_	21	UAUCAUAGUUGGUGGUUCAGG	3	1	143	115	-1.2167
a13_120551_	21	UCAACGAUAGACAUUGAUAGA	1	1	171	107	-1.2875
a12_123886_	21	UUAUCAUUGAUAGACUAGUAU	1	2	155	174	-1.0612
a11_227522_	21	CAAGCCCAUGACAAAGCAAGC	1	1	187	225	-1.0929
a14_133932_^f^	21	UCAACACGAUCGUCUAGCAUG	1	2	173	113	-1.2295
a11_203340_	21	UUUGAGUGUCCUACUCACCUC	1	1	191	411	-1.0833
**a11_11135_**	21	UAGUGCCGCGCUGCGUGCGUC	85	1	147	102	-0.98
a11_31022_	21	UUUCGCUUUUCCUCUUUCGUG	1	1	191	454	-1.1523
a12_144938_	21	UCGUGGAUAUUGCUCUUUUCU	2	1	171	504	-1.3303
a33_181157_	22	GAUAGAUACUAAUAUGCUUCUA	1	2	188	87	-1.3227
a33_37151_	22	AGAUUAAUUUAUUGGGCGUUAU	1	2	144	94	-1.3313
**a12_72169_**	22	UUGAGCUAUGCUCAGGUUGACA	30	1	176	174	-1.2933
a24_96796_^e^	22	UGAGCUAUGCUCGCUUUGGCAA	21^d^	1	175	169	-1.2855
a11_378153_	22	GAGUUCCUAAGUUUUGAUGAAU	1	1	144	351	-1.2221
a11_378297_	22	UUUUGGAUUCUAUCGAUGAAAG	1	1	155	123	-1.4857
a23_244052_^e^	22	GGGCAGCCCCACGUUGGGCAUG	5^d^	1	175	353	-0.9263
a11_85662_	22	AAAUAUAUCGGUGUCUAUCAAU	1	2	132	85	-1.2208
a21_388555_	22	GAUAGACGCUGAUAGAUAGACA	3	1	124	76	-0.8963
a11_84237_	22	CGGCCAAAAAUGACUUGCCCGG	2	1	150	105	-0.8902
a23_124460_	22	AGGUGAGUUCUUUUUAUAGGCU	1	1	184	179	-1.6306
a23_163065_	22	AUUUGAUUAGCCAAAUUUAAAC	2	2	148	130	-1.0514
a23_71826_	22	CAAUAGUCAGAUGUAAACGAUC	1	1	180	228	-1.4282
a22_52587_	22	AAAAUUAUUGGGUGAAUUAGUU	2	1	179	162	-0.9013
a13_234225_	22	UGAAUUUUGUUAUGUUUUGUAA	1	2	168	80	-2.0417
a13_286453_	22	AGUCUAUCACCGAUAGAAGCCU	1	6	182	430	-1.4372
a21_339397_	22	CGCGAGGUUCUUUGUUUGUCUU	1	1	192	413	-1.3341
a14_283014_	22	UACCUAGUGAUGCCAUUGUCAA	1	1	184	533	-1.2829
a21_170878_	22	CUAAGGUUGCCCAGAGAUGUUC	1	1	163	271	-1.2341
a21_63125_^f^	22	GAAUAAUUAUCAAGUGUGUAGC	1	1	165	210	-1.7804
a11_146182_	24	UUAAAAUGUUGCUAUAUAAUUAAU	1	6	202	390	-1.6207
a21_169735_	24	UAUACGGGCCGUAAAUAGUUUGAU	2	6	132	369	-0.8885
a23_3672_	24	UUGCUCAUUGCUAACUGCAAAGAG	1	3	198	183	-1.7594
a14_260703_^f^	24	UUAAAAGUAUGAGACGAAAAUGAA	1	1	190	111	-1.4204
a14_218837_	24	UUAAAAAGAACUACACGAACGUGC	1	1	194	342	-1.1314
a14_148530_^f^	24	AAAUAGCGUCGGGGAAAGGUGUCU	3	1	152	73	-1.0167
a21_127379_	24	UGGGACAAAAGAAAACUGUGGGUC	2	1	162	586	-1.32
a11_2899_	24	AUGAUGCUUUGGUGCUAAGGAGGU	1	1	198	470	-1.6094
a11_248538_	24	AUUUUUGGCAUUUUACACGGUGAG	2	2	192	218	-1.5206
a13_59670_	24	AGUGGAGUGGGCUAUUUUAGUCCA	6	1	166	570	-1.1249
a32_72333_	24	AACUAUUUUAUUGGAACAUGUUGA	3	1	170	96	-1.0391
a33_22103_	24	AGUAUGAUCUCGGGCUAAGGUUGC	1	4	202	246	-1.4138
a24_224684_	24	AACAAAACGAAUGAUCAAAAUGGU	3	1	134	80	-0.8871
a13_225824_	24	ACCAAAUGGAUCUAUUCUUAUAAU	1	1	198	311	-1.3393
a24_82972_	24	AACGAUCGGGUUGACUACGUAAAU	3	1	190	146	-0.9354
**a21_98074_**	24	AAUUUUUAGUGGUCCCGAAAUGCA	3	1	166	112	-0.9033
a11_350684_	24	UGAGUGUAUCAUCGAGAUAGUGCG	1	1	190	307	-1.1642
a21_216237_	24	AAAUUUCAGGGUCUAAAUUGAUGC	2	1	138	447	-1.2096
a33_49599_	24	CAGCGUGAUUGAUGGGGCAUUUUU	3	1	118	287	-1.4678
a33_58155_	24	AUGGUCGAUCUCAACCGAGAUUGA	1	1	194	298	-1.3188
a23_103110_	24	CGUGAAGAUUGUGGAUAUUGGAGA	1	1	190	414	-1.5507

^a ^Precursor secondary structures of sRNAs in bold are represented in Figure 4.

^b ^miRanda score calculated for the identification of the complementary region to the putative miRNA.

^c ^MFEI index calculated as described [35].

^d ^Include cases where sequence variants with up to two mismatches were identified and counted

^e ^Indicates miRNAs for which a miRNA* sequence was identified.

^f ^Indicates miRNAs for which an asymmetric bulge of more than 2 bases was identified in the miRNA/miRNA* duplex of the precursor, not meeting exactly the structural criteria previously set forth [36,37].

### Prediction of potential miRNA targets in the melon transcriptome

To identify potential targets of miRNAs, we screened melon unigenes in the publicly-available database [7]. Two independent searches were performed using miRanda [38] and TargetFinder [39], and the results were compared. Each program scores potential targets based on sequence complementarity, with high scores better in miRanda, and low scores better in TargetFinder. Both algorithms identified a common set of presumptive targets albeit with different scores, and the few discrepancies involved targets with low confidence scores.

Targets in Arabidopsis defined by miRanda generally have a score ≥170, and using this value as cutoff we found 150 melon unigenes as potential miRNA targets, the best of which are summarized in Table 4 (a complete list is provided in Additional file 2). The potential miRNA targets in Table 4 generally had similar annotations to their Arabidopsis counterparts, although there are some exceptions. For example, melon unigene cHS_39-F10-M13R_c is a predicted target of miR159a but it is annotated as positive regulator of brassinosteroid signaling rather than a MYB or TCP transcription factor, which is sensitive to miR159 regulation in Arabidopsis. Many of the melon unigenes identified as potential targets were not annotated, and some had previously been identified as potential miRNA precursors [5].

**Table 4:** Best quality miRNA targets identified in melon unigenes

miRNA annotation	Unigene	Score (miRanda)	Score (TargetFinder)	Unigene annotation
miR390|a, b	c15d_05-D02-M13R_c	362	2.5	non-annotated unigene
miR390|a, b	c15d_05-D02-M13R_c	362	4	non-annotated unigene
miR390|a, b	c15d_21-G08-M13R_c	362	2.5	non-annotated unigene
miR390|a, b	c15d_21-G08-M13R_c	362	4	non-annotated unigene
miR391|a	cCL286Contig1	325	--	histone H1, putative
miR164|a, b	cA_04-D07-M13R_c	319	--	non-annotated unigene
miR390|a, b	cCL384Contig1	316	--	ATSK11, SK 11ATSK11; protein kinase/protein serine/threonine kinase
miR391|a	cPSI_29-G09-M13R_c	313	--	UVR8UVR8 (UVB-RESISTANCE 8); chromatin binding/guanyl-nucleotide exchange factor
miR167|d	cCL1653Contig1	200	--	non-annotated unigene
miR167|d	cCL2516Contig1	200	--	non-annotated unigene
miR167|a, b	cCL1653Contig1	195	1	non-annotated unigene
miR167|a, b	cCL2516Contig1	195	0	non-annotated unigene
miR168|a, b	c46d_19-A03-M13R_c	195	0	non-annotated unigene
miR171|a	cHS_39-C12-M13R_c	195	0	scarecrow-like transcription factor 6 (SCL6)
miR397|a	c15d_32-E08-M13R_c	195	0	potential miR397a precursor
miR160|a, b, c	cCL5073Contig1	191	0.5	ARF17ARF17 (AUXIN RESPONSE FACTOR 17); transcription factor
miR164|a, b	cPSI_18-H09-M13R_c	190	3	ANAC100, ATNAC5ANAC100 (ARABIDOPSIS NAC DOMAIN CONTAINING PROTEIN 100)
miR393|a, b	cCL3757Contig1	190	2	AFB2AFB2 (AUXIN SIGNALING F-BOX 2); auxin binding/ubiquitin-protein ligasechr3
miR393|a, b	cCL4853Contig1	190	1	TIR1TIR1 (TRANSPORT INHIBITOR RESPONSE 1); auxin binding/protein binding/ubiquitin-protein ligase
miR408|a	cCL975Contig1	190	2.5	ARPNARPN (PLANTACYANIN); copper ion binding/electron carrierchr2
miR408|a	cHS_18-D07-M13R_c	190	2.5	ARPNARPN (PLANTACYANIN); copper ion binding/electron carrierchr2
miR157|a, b, c	c46d_26-C05-M13R_c	187	3	SPL4SPL4 (SQUAMOSA PROMOTER BINDING PROTEIN-LIKE 4); DNA binding/transcription factor
miR157|a, b, c	cCI_30-A09-M13R_c	187	2	SPL9SPL9 (SQUAMOSA PROMOTER BINDING PROTEIN-LIKE 9); transcription factor
miR157|a, b, c	cCL2877Contig1	187	3	SPL3SPL3 (SQUAMOSA PROMOTER BINDING PROTEIN-LIKE 3); DNA binding/transcription factor
miR164|a, b	cCI_64-A04-M13R_c	187	2	NAC1, ANAC022NAC1; transcription factorchr1
miR170|a	cHS_39-C12-M13R_c	187	1.5	scarecrow-like transcription factor 6 (SCL6)
miR159|a	cHS_39-F10-M13R_c	183	3	brassinosteroid signaling positive regulator-related
miR161|a.2	cA_16-D06-M13R_c	183	3	pentatricopeptide (PPR) repeat-containing protein
miR169|a	cA_37-E12-M13R_c	183	4	NF-YA9NF-YA9 (NUCLEAR FACTOR Y, SUBUNIT A9); specific transcriptional repressor/transcription factor
miR156|a, b, c, d, e, f	c46d_26-C05-M13R_c	182	2	SPL4SPL4 (SQUAMOSA PROMOTER BINDING PROTEIN-LIKE 4); DNA binding/transcription factor
miR156|a, b, c, d, e, f	cCI_30-A09-M13R_c	182	1	SPL9SPL9 (SQUAMOSA PROMOTER BINDING PROTEIN-LIKE 9); transcription factor
miR156|a, b, c, d, e, f	cCL2877Contig1	182	2	SPL3SPL3 (SQUAMOSA PROMOTER BINDING PROTEIN-LIKE 3); DNA binding/transcription factor
miR157|d	c46d_26-C05-M13R_c	182	3	SPL4SPL4 (SQUAMOSA PROMOTER BINDING PROTEIN-LIKE 4); DNA binding/transcription factor
miR157|d	cCI_30-A09-M13R_c	182	2	SPL9SPL9 (SQUAMOSA PROMOTER BINDING PROTEIN-LIKE 9); transcription factor
miR157|d	cCL2877Contig1	182	3	SPL3SPL3 (SQUAMOSA PROMOTER BINDING PROTEIN-LIKE 3); DNA binding/transcription factor
miR319|a, b	c15d_24-H05-M13R_c	182	4	potential miR319|a, b precursor
miR167|d_melon	cCL1653Contig1	--	0	non-annotated unigene
miR167|d_melon	cCL2516Contig1	--	0.5	non-annotated unigene
miR172|e	c15d_13-C08-M13R_c	--	2	non-annotated unigene
miR172|e	cA_04-D07-M13R_c	--	2	non-annotated unigene
miR167|a, b	cCL2288Contig1	--	2.5	unknown protein
miR156|a, b, c, d, e, f	cCL5542Contig1	173	2.5	kelch repeat-containing F-box family protein
miR157|a, b, c	cCL2547Contig1	175	2.5	unknown protein
miR164|a, b	cCL2655Contig1	175	2.5	non-annotated unigene

**Figure 5:**
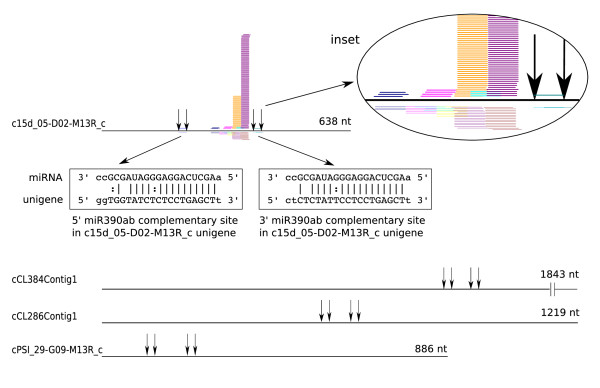
**Identification of potential ta-siRNA transcripts**. Four unigenes were found to have two miRNA target sites each. For each unigene, the region delimited by the two miRNA suites was used as a BLAST query against the sRNA data set and only c15d_05-D02-M13R_c generated hits. Unigenes are represented by black horizontal lines. The miRNA site boundaries are represented by vertical arrows. Potential ta-siRNAs are represented in the inset by colored short horizontal lines mapped onto the unigene.

Interestingly, the highest miRanda scores (> 300) were achieved for transcripts with two separate miRNA targets on the same molecule. For example, unigene c15d_05-D02-M13R_c had two target sites for miR390ab separated by ~200 nt. When this region was used as a BLAST query against the melon sRNA dataset, a group of 257 sequences (more than 92% of them being 21 nt long) was identified with nearby clusters of related 21-nt sequences in both the sense and antisense orientations, which is reminiscent of the ta-siRNAs biogenesis mechanism [18] (Figure 5). Both sites (complementary to miR390 family members in unigene c15d_05-D02-M13R_c) had similar miRanda scores, they did not contain mismatches or G:U wobbles involving nucleotides 9-11 and were phased 21 nt one of each other. The number of sRNA copies was different in each cluster and were more abundant in sense orientation compared to antisense orientation. Two registers of phased 21-nt siRNAs were observed. One of them was phased with the miR390 complementary sites but the other one was not. A representative sequence from each cluster was selected and used to search for potential targets in melon unigenes, identifying > 100 transcripts with a miRanda score > 170. Several of these transcripts were annotated as ARFs and ubiquitin related gene products (Table 5).

**Table 5:** miRNA targets identified in melon transcripts for potential ta-siRNAs derived from unigene c15d_05-D02-M13R_c

Melon sRNA name	sRNA sequence	Targeted unigene	Unigene sense	Score (miRanda)	Unigene annotation
a11_156739_	AGTTTGCTTCTTGGGCTCTTC	cA_05-B09-M13R_c	Forward	175	IAA16; transcription factor
a11_156739_	AGTTTGCTTCTTGGGCTCTTC	cPS_07-G03-M13R_c	Forward	175	IAA16; transcription factor
a14_55988_	AGAGCCCAAGAAGCAAACTGG	cCL678Contig1	Forward	172	auxin efflux carrier family protein
a24_92242_	AGAGCCCAAGAAGCAAACTG	cCL678Contig1	Forward	172	auxin efflux carrier family protein
a33_151240_	CAGTTTGCTTCTTGGGCTCTT	c15d_39-H01-M13R_c	Forward	171	ARF6 (AUXIN RESPONSE FACTOR 6); transcription factor
a14_362833_	CGATGGTGATGGGATTTTTGA	cCL1479Contig1	Reverse	171	IAA9 (INDOLE-3-ACETIC ACID INDUCIBLE 9); transcription factor
a14_362833_	CGATGGTGATGGGATTTTTGA	cCL4756Contig1	Reverse	175	ATAUX2-11 (AUXIN INDUCIBLE 2-11); DNA binding/transcription factor
a14_362833_	CGATGGTGATGGGATTTTTGA	cP5.72_c	Reverse	175	IAA7 (INDOLE-3-ACETIC ACID 7); transcription factor
a33_203464_	CATTTTTTACGATGGTGATGG	cCL3310Contig1	Forward	179	ATUBP3 (ARABIDOPSIS THALIANA UBIQUITIN-SPECIFIC PROTEASE 3)
a14_362833_	CGATGGTGATGGGATTTTTGA	cCL3310Contig1	Forward	175	ATUBP3 (ARABIDOPSIS THALIANA UBIQUITIN-SPECIFIC PROTEASE 3)
a14_20904_	TACGATGGTGATGGGATTTTT	cCL4210Contig1	Forward	174	ubiquitin-associated (UBA)/TS-N domain-containing protein
a11_156739_	AGTTTGCTTCTTGGGCTCTTC	cCL1290Contig1	Forward	171	binding/ubiquitin-protein ligase

The remaining unigenes with two predicted miRNA target sites listed in Table 4 were annotated as protein-coding transcripts and no sRNAs were identified with similarity to the region flanked by the two target sites (Figure 5), suggesting that they did not account for authentic ta-siRNA-producing loci. Targets were also sought in the reverse-complement sequences of melon unigenes, because a small proportion of the ESTs could be incorrectly oriented as an artifact of the cloning procedure [5]. Twenty-eight unigenes were identified as potential miRNA targets using the same criteria described above, most of which were found to be non-annotated (Table 6). In this new set of data, unigenes with two targets were used again as a BLAST query against the melon sRNA dataset but no hits were obtained, so these unigenes were no longer considered as potential ta-siRNAs.

**Table 6:** Best quality miRNA targets identified in reverse-complement sequences of melon unigenes

miRNA annotation	Unigene	Score (miRanda)	Score (TargetFinder)	Annotation
miR167|d	cCI_22-D03-M13R_c	327	--	metalloendopeptidase
miR390|a, b	cCL2179Contig1	317	--	ARAC1, ATGP2, ATRAC1, ROP3, ATROP3 | ARAC1; GTP binding
miR167|d	cPSI_41-B02-M13R_c	200	--	non-annotated unigene
miR157|a, b, c	cCI_04-H02-M13R_c	195	0	non-annotated unigene
miR166|a, b, c, d, e, f, g	cA_31-D02-M13R_c	195	0	non-annotated unigene
miR166|a, b, c, d, e, f, g	cCI_54-H07-M13R_c	195	0	non-annotated unigene
miR166|a, b, c, d, e, f, g	cCI_69-H04-M13R_c	195	1	non-annotated unigene
miR167|a, b	cPSI_41-B02-M13R_c	195	1	non-annotated unigene
miR168|a, b	cCI_38-C07-M13R_c	195	0	non-annotated unigene
miR170|a	cPSI_40-F10-M13R_c	191	0.5	non-annotated unigene
miR397|a	c46d_36-B03-M13R_c	191	1.5	LAC10 (laccase 10); laccase
miR157|d	cCI_04-H02-M13R_c	190	0	non-annotated unigene
miR171|b, c	cPSI_40-F10-M13R_c	190	1	non-annotated unigene
miR319|a, b	c15d_24-H05-M13R_c	190	0	non-annotated unigene
miR165|a, b	cA_31-D02-M13R_c	187	1	non-annotated unigene
miR165|a, b	cCI_54-H07-M13R_c	187	1	non-annotated unigene
miR165|a, b	cCI_69-H04-M13R_c	187	2	non-annotated unigene
miR171|a	cPSI_40-F10-M13R_c	187	2	non-annotated unigene
miR159|a	cCL1409Contig2	183	3	brassinosteroid signaling positive regulator-related
miR159|b	cCL1409Contig2	183	4	brassinosteroid signaling positive regulator-related
miR159|c	cCL1409Contig2	183	4	brassinosteroid signaling positive regulator-related
miR169|a	c15d_10-G06-M13R_c	183	4	non-annotated unigene
miR169|b, c	c15d_10-G06-M13R_c	183	4	non-annotated unigene
miR169|h, i, j, k, l, m, n	c15d_10-G06-M13R_c	183	3	non-annotated unigene
miR156|a, b, c, d, e, f	cCL1781Contig1	181	3	DNA-directed RNA polymerase II, putative (RPB10)
miR167|d_melon	cPSI_41-B02-M13R_c	--	0	non-annotated unigene
miR159|c	c15d_24-H05-M13R_c	--	2	non-annotated unigene
miR159|b	c15d_24-H05-M13R_c	--	2.5	non-annotated unigene

With some exceptions, several miRNA targets with miRanda scores ≥170 (see Additional File 3) were identified for each of the potential novel melon-specific miRNAs listed in the previous section.

### Characterization of other melon sRNAs

**Figure 6:**
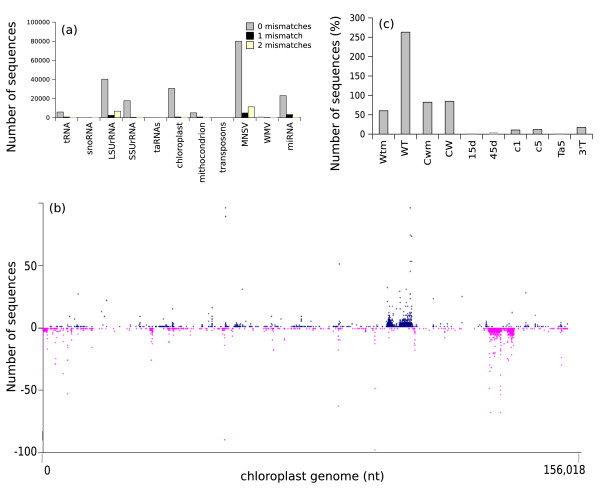
**Types and frequencies of known melon sRNAs**. *(a) *Number of sRNAs with similarity to known sequences in public databases: tRNA = transfer RNA; snoRNA = small nucleolar RNA; LSUrRNA = large subunit of ribosomal RNA; SSUrRNA = small subunit of ribosomal RNA; taRNA = *trans*-acting-si-RNAs; transposon = transposon sequences; chloroplast = melon chloroplast genome; mitochondrion = melon mitochondrial genome; MNSV = *Melon necrotic spot virus *genome; WMV = *Watermelon mosaic virus *genome; miRNA = microRNA. *(b) *Mapping of small RNAs onto the melon chloroplast genome. X-axis represents genome nucleotide positions. Y-axis represents the number of sRNAs mapped at each position. The chloroplast genome is represented by a horizontal black line (156,018 nt in length) at y = 0, and the 5' ends of sRNAs are represented by points (blue: sense sRNAs, pink: antisense sRNAs). The frequencies of antisense sRNAs are shown as negative numbers. *(c) *Number of melon sRNAs with similarity to the melon chloroplast genome.

Next, we blasted our sRNA sequences against RNA and genomic databases to search for other sRNA species by sequence similarity (Figure 6). sRNAs similar to transfer RNA (tRNA), trans-acting siRNA, small nucleolar RNA (snoRNA) and transposons were the least abundant, whereas ribosomal RNAs (rRNA) were largely the most abundant non-coding sRNA species (Figure 6A). Intriguingly, exogenous virus-derived sRNAs were as abundant as other endogenous plant sRNAs, at least in the case of MNSV. Most of the sRNAs identified had complete sequence similarity with the reference RNA from each database (Figure 6A), even if up to two mismatches were allowed in BLAST comparisons. The only exception were sequences similar to ta-siRNAs, for which 14 melon sRNAs with similarity to Arabidopsis TAS3a|D7(+) and TAS3a|D8(+) were identified, 2 containing 1 mismatch, and 12 containing 2 mismatches. All of them mapped very close in the melon genome and in a different region than c15d_05-D02-M13R_c unigene (the other potential source of ta-siRNAs, see above). To determine if they were authentic melon ta-siRNAs, we selected a 600 bp window sequence upstream and downstream from the genomic location determined in the melon genome for each candidate; then, a BLAST query against the melon sRNA dataset was performed, revealing that at least 126 sequences (95 of them being 21-nt in length) mapped in this region and were arranged according to a near 21-nt phase spacing (data not shown).

Many sRNA sequences also generated hits in the plastid genomes (30,239 sRNAs corresponding to 4,254 unique plastid sequences). When these sRNAs were mapped onto the melon chloroplast genome (unpublished data) (Figure 6B), two clusters of sequences resolved in regions presumably annotated as chloroplast rRNA. These regions lie within two inverted genomic repeats, and sRNAs were accordingly identified in both the sense and antisense orientations. Some of the chloroplast sRNAs had previously been cloned in other species [40]. For example, melon sRNA a33_398374 (sequence AGT TAC TAA TTC ATG ATC TGG C) was the most abundant melon plastid sRNA (18,054 counts), and a matching sequence is present in more than 900 chloroplast genomes. It is located in an intergenic region and may target a methyltrasferase transcript, although there is no direct evidence that it has silencing functions. Melon sRNA a14_392967_ (sequence GGT AGT TCG ATC GTG GAA TTT) was less abundant (166 counts), it is present in 10 different chloroplast genomes, and it may target a transcript encoding an electron carrier protein. Interestingly, different numbers of plastid sequences were obtained from each library (Figure 6C). For example, in the virus-resistant melon accession TGR-1551 there was no difference in the number of sRNAs with hits to melon chloroplast genome between healthy and virus inoculated samples, but in the virus-susceptible accession Tendral, more sRNAs were counted in inoculated samples (Figure 6C).

Unlike chloroplast sRNAs, only 7,854 sRNAs (corresponding to 2,384 unique sequences) matched the melon mitochondrial genome (unpublished data). These sRNAs were mapped on the mitochondrial genome sequence and formed three clusters, again corresponding to the sites of rRNA genes (data not shown).

## Discussion

In this report, we describe the first screen for melon sRNAs by deep sequencing. In total, 398,450 high-quality sequences were generated, representing 90% of the total raw reads. RNA species 21, 24, 20 and 22 nt in length dominated the sRNA transcriptome in melon with the 21-nt class being the most abundant in our libraries. Molecules of 24-nt processed by DCL3 are often the most abundant endogenous plant sRNAs [13], but this may vary according to species. For example, 24-nt sRNAs are more abundant in Arabidopsis, rice and tomato [41,42,9], whereas 21-nt sRNAs are more abundant in grapevine, wheat and conifers [12,43,44]. It is also possible that the composition of the sRNA population of a given plant species varies according to tissue and physiological conditions, as seems to be the case of melon (see Additional file I). Perhaps the higher proportion of 24-nt sRNAs found in melon ovaries compared to the other tissues reflects the predominance of developmental processes based on epigenetic events in the ovary.

Recent studies have shown that *cis*-acting siRNAs arising from heterochromatin, transposons and other repeat elements account for the greatest proportion of endogenous sRNA populations in plants [13,45-48]. In melon, only ~7,000 sRNAs matched known transposon sequences, in contrast to ~60,000 sRNA sequences matching ribosomal RNA, which may simply reflect the paucity of melon transposon sequence information in databases, as only 1.5% of the melon genome has been annotated for transposable elements [49]. Transposon sequences in different species show more divergence than rRNA sequences, so the representation of transposon-related sRNAs could increase when a more accurate and complete annotation of the melon genome becomes available. We also identified two sets of ta-siRNAs in our data, which mapped to different loci in the melon genome thus revealing the presence of at least two potential *TAS *genes. One locus was not represented in the melon unigene database, most likely because of its incomplete coverage. The sequence of the other locus was similar to that of a non-annotated melon transcript, and contained two registers of sRNAs in a 21-nt phase bounded by two target sites to miR390ab, reminiscent to *TAS3 *genes. Non-coding transcripts containing two miR390 complementary sites that give rise to phased siRNAs have been described in other organisms. In the moss *Physcomitrella patens*, both 3' and 5' target sites are cleaved. In Arabidopsis, the 5' miR390 complementary site contains a mismatch and two G:U wobbles involving positions 9-11 and, despite it is not cleaved, it binds the silencing complex and is required for full *AtTAS3 *function *in vivo *[50]. In melon, both 3' and 5' miR390 had perfect complementarity at positions 9-11, suggesting that both could be cleaved, as opposed to Arabidopsis, to specify a phased register for ta-siRNA biogenesis. Interestingly, an additional siRNA register that is likely independent of miR390-directed cleavage of the putative melon TAS transcript was observed. This alternative register might be determined by the processing activity of TAS transcripts by one of the most abundant melon primary ta-siRNAs during generation of secondary ta-siRNAs (Figure 5), as proposed for alternatively phased *TAS3 *ta-siRNAs in Arabidopsis [18,50]. Since there are additional *TAS *loci in other plant genomes, it is reasonable that other melon *TAS *loci remain to be discovered.

More than 30,000 of our sRNA sequences matched the plastid genome, suggesting intense sRNA activity in this organelle. Mitochondrion-specific sRNAs were less abundant in comparison. The abundance of plastid sRNAs varied by source, with fewer sequences obtained from the ovary and fruit libraries compared to the cotyledon libraries, perhaps reflecting a relationship between chloroplast sRNA activity and photosynthesis. Interestingly, there was no significant difference in sRNA accumulation when comparing infected and healthy TGR-1551 cotyledons (resistant to WMV) whereas more sRNA accumulated in healthy Tendral (susceptible to WMV and MNSV) cotyledons than in infected ones. Whether or not this is related with the resistance phenotype is a matter of speculation.

More than 28,000 melon sRNAs in our sequenced collections matched known miRNAs in other plants, and 46 distinct melon sRNA species could be assigned to 26 known miRNA families. Although we generated a relatively low number of sequence reads, our data nevertheless were in good harmony with previous studies of miRNA profiling based on exhaustive sequencing of sRNA populations (e.g. in grapevine, 24 million reads, 26 known miRNA families and 26 non-conserved miRNA families; in tomato, 721,874 reads, 30 known miRNA families; and in orange, 13,106,573 reads, 42 highly-conserved miRNA families) [12,11,9]. This probably reflects the generally-accepted high level of expression reported for conserved miRNAs.

In addition to known miRNAs, 84 sRNA sequences derived from genomic loci with intramolecular folding capacities and not previously described as miRNAs in other plant species were predicted as potential melon-specific miRNAs. In most cases, only one sequence was counted from each of these miRNAs, which is consistent with reports suggesting that species-specific miRNAs are usually expressed at low level and in a tissue-specific manner [41]. The candidates listed in Table 2 include a number of special cases, i.e. miRNAs with miRanda scores ≥195 and very strong secondary structures including an internal loop, resembling type III foldback transposons [51,52,9]. Although these sequences do not match known melon transposons, they were not considered as miRNA candidates because accurate homology-based transposon annotation and prediction occasionally needs to be complemented with *ab initio *approaches based on structural features [53,49]. However, even not considering this particular group, our data indicate that most of the precursors we identified are candidates to encode melon-specific miRNAs.

The accumulation of miRNAs was estimated by census sequencing and this showed that there is more miRNA diversity and that miRNAs are more abundant in ovaries than fruits. Although miRNAs are involved in many processes, 60-70% of known plant miRNAs control the expression of transcription factors that regulate critical developmental processes, such as proper specification of floral organ identity or leaf polarity, and over-expression or knockout of *MIRNA *genes led to severe developmental defects [48,54,13]. It is likely that the greater abundance of miRNAs in the early ovary stages compared to fruit reflects the more significant developmental activity in ovaries, and confirms that meristems and other developmentally active tissues are good resources for miRNA screening.

The comparison of healthy and virus-infected melon tissues showed that generally miRNAs were less abundant in infected tissues. Viruses interfere with and exploit endogenous RNA-silencing pathways using diverse strategies [55,19]. For example, the potyvirus silencing suppressor HC-Pro has been shown to suppress the miRNA pathway by inhibiting miRNA assembly into AGO1-containing silencing complexes and unwinding of miRNA/miRNA* duplexes, causing accumulation of stable duplexes [56]. Several studies have shown that virus infection can regulate the accumulation of mRNAs targeted by miRNAs without affecting the abundance of the miRNAs themselves, or even by promoting a slight accumulation [57-59]. In contrast, we found that miRNA accumulation was generally depressed in infected plants compared to controls subjected to mock inoculations. A notable exception was miR168ab, which was upregulated in the resistant genotype but downregulated in the susceptible one. This miRNA has previously been shown to be involved in controlling the expression of ARGONAUTE1 (AGO1), the catalytic subunit of the RNA-induced silencing complex responsible for slicing of target mRNAs [60]. Recent work has described the enhanced expression of miR168 and *AGO1 *mRNA in virus-infected plants specifically and independently of other miRNAs [61-63]. The contrasting miRNA profiles observed in the TGR-1551 and Tendral varieties suggests that silencing may underly the resistance of TGR-1551 to WMV, although this is a hypothesis that will require further research.

We have identified more than 150 melon unigenes as potential targets for the known and novel miRNA sequences discovered in this investigation. Many animal transcripts are targets for more than one miRNA but this phenomenon is uncommon in plants [64]. Accordingly, most of melon unigenes identified as potential targets featured only a single miRNA site. miRNAs that are conserved across species tend to have conserved targets too, and our data confirm this is the case in melon. However, several unigenes predicted with high confidence as targets for conserved miRNAs had different annotations to the corresponding target genes in Arabidopsis, although these may represent false positives that would fail additional validation. Furthermore, the non-conserved targets of conserved miRNAs can be cleaved at a lower frequency than conserved targets [12]. For these two reasons, the selection of targets for individual validation experiments can be challenging.

An interesting alternative for miRNA target discovery in a genome-scale is the analysis of the small RNA degradome [65], as this avoids the *a priori *selection of potential targets. High-throughput gene expression profiling techniques such as microarray hybridization can also help to predict miRNA targets because some times a negative correlation between the abundance of miRNAs and their target mRNAs can be identified [66,67]. We have used microarrays to monitor gene expression profiles in healthy TGR-1551 and Tendral plants and plants infected with WMV. When compared with our miRNA data, we were able to identify two unigenes encoding AGO proteins that were differentially expressed and showed contrasting expression profiles in susceptible and resistant genotypes (Gonzalez-Ibeas and Aranda, unpublished data). The same profile was observed for miR168 accumulation, suggesting that miR168 may be involved in virus resistance and providing the basis for future experiments.

## Conclusion

We have analysed and catalogued a collection of melon endogenous sRNA obtained through massive cDNA sequencing and have identified known miRNAs and ta-siRNAs (conserved in other species) as well as potential melon-specific miRNAs with no database matches. We have also identified potential targets for these miRNAs in the melon transcriptome. Census sequencing (i.e. counting the number of sequence reads for each sRNA) was used to profile their expression in different tissues, and in healthy *vs*. virus-infected cotyledons. By comparing the predicted targets and the differential expression profiles we were able to provide insights into the role of miRNAs in the regulation of fruit development and plant-virus interactions.

## Methods

### Plant material

Small RNA libraries were prepared using material from three melon accessions: 1) the Tendral cultivar (Semillas Fitó, Barcelona, Spain), which is susceptible to MNSV and WMV, 2) the breeding line T-111 of the cultivar Piel de Sapo (Semillas Fitó, Barcelona, Spain), and 3) the genotype TGR-1551 (germplasm collection of "Estación Experimental La Mayora" (EELM-CSIC), Málaga, Spain), which is resistant to WMV.

Melon plants were grown under greenhouse conditions (~25/20°C, 16-h photoperiod, ~70% relative humidity) in 0.5-L pots with substrate (Tendral and TGR-1551) or in soil bags with the capacity for four plants (Piel de Sapo). Fruits of 15 and 45 days after pollination (DAP) were collected and mesocarp tissues were recovered and used for RNA extractions. Virus infected samples were obtained from completely expanded cotyledons rubbed with carborundum (ø = 0.037 mm) and the corresponding viral inoculum. MNSV-infected melon cotyledons exhibiting lesions and marrow leafs systemically infected with WMV were ground in cold inoculation buffer (0.2 M phosphate buffer pH = 8.0, 0.1% (v/v) *beta*-mercaptoethanol, 0.03 g/ml activated charcoal) for inoculum preparation. Mock-inoculated control cotyledons were rubbed with inoculation buffer and carborundum alone.

Cotyledons were harvested at 1, 3, 5 and 7 days post-inoculation (dpi) and pooled for RNA extraction. Fruit samples were prepared as previously described [8]. Ovaries were collected at stages C1 and C5 (Mascarell-Creus et al., unpublished). The C1 stage corresponds to flower emergence from the inflorescence bud, when the outermost perianth organs commence development and no floral whorls are visible. The C5 stage corresponds to anthesis, when the flower is ready to be fertilized and all floral organs are fully formed, including the yellow petals that attract pollinators. Under normal growth conditions, C1 to C5 development takes approximately 5 days.

### Small RNA library construction

Total RNA was extracted using Trizol-Reagent (Sigma Chemical Co., St. Louis, MO, USA) and 300 μg were used to construct sRNAs libraries as described [57,32]. The 3' adaptor was replaced with a pre-activated 5'-adenylated oligonucleotide (5'-rAppCT GTA GGC ACC ATC AAT 3ddC-3') (Integrated DNA Technologies, Coralville, Iowa, USA) to avoid sRNA circularisation.

Ten chimeric RNA/DNA oligonucleotide 5' adaptor variants were generated by modifying the four-nucleotide identifier (barcode): 1-1, ATC GTA GGC ACC UGA UA; 1-2, ATC GTA GGC CAC UGA UA; 1-3, ATC GTA GGC UGC UGA UA; 1-4, ATC GTA GGC GUC UGA UA; 2-1, ATC GTA GCG ACC UGA UA; 2-2, ATC GTA GCG CAC UGA UA; 2-3, ATC GTA GCG UGC UGA UA; 2-4, ATC GTA GCG GUC UGA UA; 3-1, ATC GTA GAC GCC UGA UA; 3-2, ATC GTA GAC CGC UGA UA. After each ligation step, sRNA was purified by 17% denaturing polyacrylamide gel electrophoresis. The purified, ligated sRNA was reverse transcribed with SuperScript^® ^III reverse transcriptase (Invitrogen BV/Novex, Groningen, Netherlands) and the cDNA was amplified with AmpliTaq Gold^® ^DNA Polymerase (Applied Biosystems, Foster City, CA, USA) using 3' PCR FusionB and 5' PCR FusionA primers [57]. The PCR primers contained the "A" and "B" tag sequences compatible with 454 technology [31].

DNA amplicons were gel-purified using 4% Metaphor Agarose and isolated using the QIAEX II Gel Extraction Kit (Qiagen, Hilden, Germany). The quantity and quality of the DNA amplicons were determined using a ND-1000 spectrophotometer (NanoDrop Technologies, Wilmington, Delaware, USA) and an Experion Automated Electrophoresis System (Bio-Rad, Hercules, California, USA). The same quantity of DNA from each library was pooled and sequenced using the 454 Life Science Technology platform (Lifesequencing S.L., Paterna, Valencia, Spain). Sequence data in this publication have been deposited in NCBI's Gene Expression Omnibus and are accessible through GEO Series accession number GSE28653 http://www.ncbi.nlm.nih.gov/geo/query/acc.cgi?acc=GSE28653.

### Bioinformatics

The sRNA sequences were parsed from FASTA-formatted files containing 447,180 reads from two independent 454 sequencing runs and assigned to specific libraries by identifying the sRNA/adaptor boundaries and barcode analysis. Sequences were analysed with standard Python scripts [68] and the BioPython library [69]. Only sequences with the 3' and 5' adaptors in the correct position were considered. Known sRNAs were identified by searching public databases using BLAST version 2.2.19 [70] and allowing up to two mismatches. The following databases and sequences were searched: Transfer RNA Database (version 2009) [71], Plant Small Nucleolar RNA Database (v1.2) [72], SILVA (ribosomal RNA database, v100) [73], The Arabidopsis Small RNA Project (ASRP) Database [33], Rfam Database 10.0 [74], miRBase (release 16) [34], The Plant Repeat Database [75], *Cucumis melo *chloroplast genome (unpublished data), *Cucumis melo *mitochondrial genome (unpublished data), MNSV genome (GenBank accession AY122286.1), WMV genome (GenBank accession AY437609.1). In the case of miRNAs, melon sequences were named with the reference miRNA from each database in order to distinguish miRNA species of each family. miRNA targets were identified using miRanda v3.0 [38] and TargetFinder Perl script 1.5 [39]. Putative novel melon-specific miRNA genes were identified by using the candidate miRNA as a BLAST query against the melon genome (unpublished data). For each hit, 600 bp of sequence upstream and downstream of the alignment was used to search for a near-perfect reverse complement (miRNA*) sequence with the miRanda algorithm. Regions lacking a corresponding miRNA* sequence were discarded. Minimum genomic regions (> 70 nt) containing miRNA and miRNA* sequences were selected as potential precursors. Those corresponding to protein-coding genes were identified by BLAST searches against the Arabidopsis protein database (TAIR) and were discarded, whereas non-coding potential precursors were manually inspected and used to predict the RNA secondary structure with Mfold [76] and for calculation of the MFEI index [35]. Precursors that met structural miRNA criteria were selected for further evaluation [36,37].

## Authors' contributions

DGI prepared RNA from infected and mock-inoculated samples, constructed all the sRNA libraries, carried out the trimming and analysis of the sRNA sequences and wrote the manuscript. JB provided bioinformatics analysis support. LD and CL provided guidance for the preparation of the sRNA libraries and additional technical support. ACD, AMC, MS and JGM prepared RNA from fruit and ovary samples. MAA supervised DGI including writing of the manuscript, and conceived this study together with DGI, JGM and CL. All authors read and approved the final manuscript.

## Supplementary Material

Additional file 1 **Length distribution of the small RNA data set for each library**. Length distribution of melon sRNAs for each library (listed in Table 1). Sequence numbers are shown as a percentage of the total number of sequences obtained from every library. Data are given for total (with redundancy) and unique (no redundancy) sequences. Click here for file

Additional file 2 **Known miRNA targets identified in melon unigenes**. Complete set of all known miRNA targets identified in melon unigenes. Click here for file

Additional file 3 **Novel melon-specific miRNA targets identified in melon unigenes**. Complete set of all novel melon-specific miRNA targets identified in melon unigenes. Click here for file
