# Supplementary figures and images for: Analysis of the melon (Cucumis melo) small RNAome by high-throughput pyrosequencing
Source: BMC Genomics. 2011 Aug 3;12:393. doi: 10.1186/1471-2164-12-393 (PMC3163571; doi:10.1186/1471-2164-12-393)

Library

Total sequences

Unique sequences

WTm

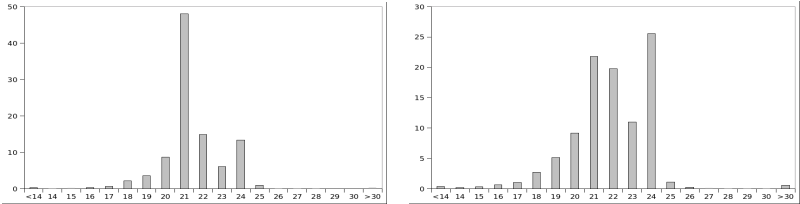

WT

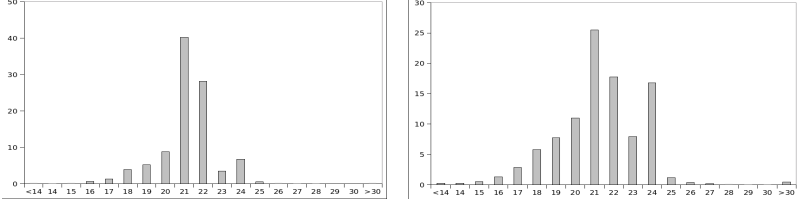

CWm

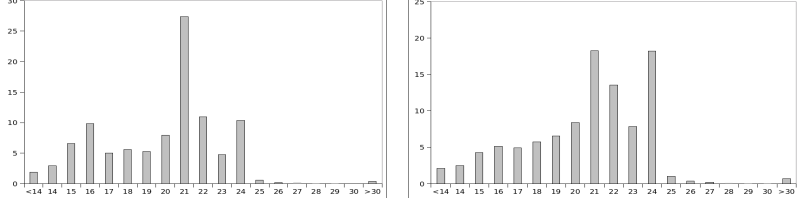

CW

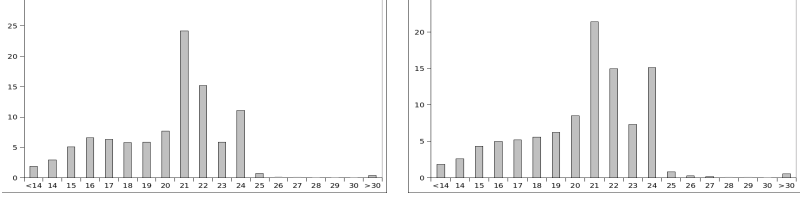

15d

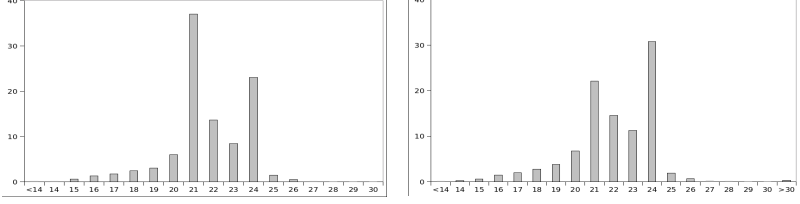

45d

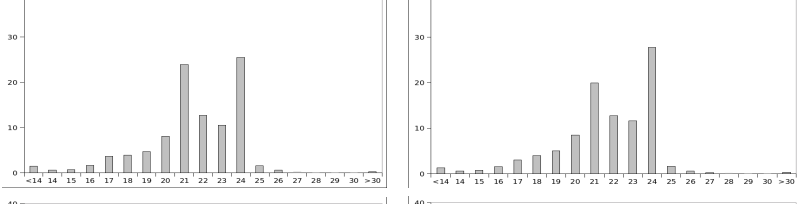

C1

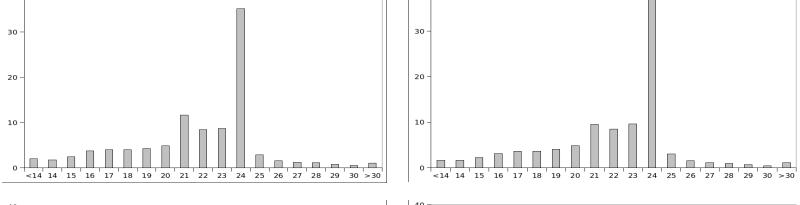

C5

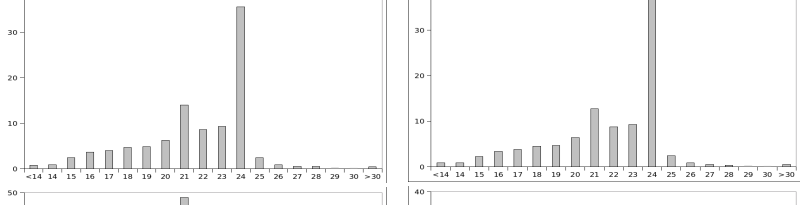

Ta5

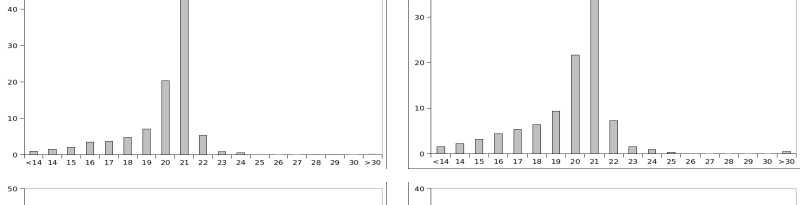

3'T

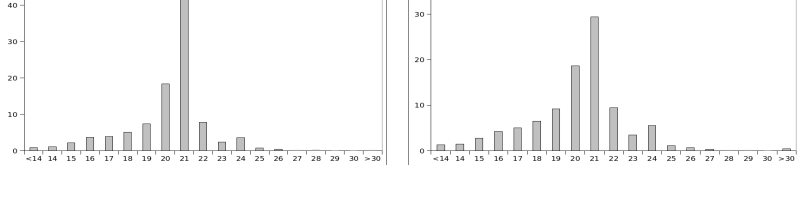

Supplement: Additional file 1 — Length distribution of the small RNA data set for each library. Length distribution of melon sRNAs for each library (listed in Table 1). Sequence numbers are shown as a percentage of the total number of sequences obtained from every library. Data are given for total (with redundancy) and unique (no redundancy) sequences. [file 1471-2164-12-393-S1.PDF]
